# Evaluation of plasma nucleosome concentrations in dogs with a variety of common cancers and in healthy dogs

**DOI:** 10.1186/s12917-022-03429-8

**Published:** 2022-08-31

**Authors:** H. M. Wilson-Robles, T. Bygott, T. K. Kelly, T. M. Miller, P. Miller, M. Matsushita, J. Terrell, M. Bougoussa, T. Butera

**Affiliations:** 1 College of Veterinary Medicine, Small Animal Clinical, Sciences Department, Texas A&M University, College Station, TX 77843 USA; 2 Volition America & Volition Veterinary Diagnostic Development, 13215 Bee Cave, Parkway, Galleria Oaks B, Suite 125, Austin, Texas 78738 USA; 3 Volition Diagnostics UK Ltd, 93-95 Gloucester Place, London, W1U 6JQ UK; 4 Belgian Volition SRL, ParcScientifiqueCrealys, 22 Rue Phocas Lejeune, 5032 Isnes, Belgium

**Keywords:** Nucleosome, Histone, H3.1, Canine, Cancer, Osteosarcoma, Mast cell tumor, Histiocytic sarcoma, Melanoma, Soft tissue sarcoma

## Abstract

**Background:**

Cell free DNA, in the form of nucleosomes, is released into circulation during apoptosis and necrosis in a variety of diseases. They are small fragments of chromosomes that are composed of DNA wrapped around a histone core made of four duplicate histone proteins forming an octamer. The nucleosome compartment is a relatively uninvestigated area of circulating tumor biomarkers in dogs. The objectives of this study were to quantify and better characterize nucleosome concentrations in 528 dogs with various common malignancies and compare them to 134 healthy dogs.

**Results:**

The sensitivity of increased circulating nucleosome concentrations for the detection of cancer in all dogs was 49.8% with a specificity of 97% with an area under the curve of 68.74%. The top 4 malignancies detected by the test included lymphoma, hemangiosarcoma, histiocytic sarcoma and malignant melanoma. The malignancies least likely to be detected were soft tissue sarcomas, osteosarcoma and mast cell tumors.

**Conclusions:**

A variety of tumor types may cause increased nucleosome concentrations in dogs. Tumors of hematopoietic origin are most likely to cause elevations and local tumors such as soft tissue sarcomas are least likely to cause elevations in plasma nucleosome concentrations.

## Background

Biomarkers for the early detection of cancer have revolutionized cancer screening in healthy and high-risk human populations. These cancer screening tests enable enhanced opportunities for earlier treatment and higher cure rates in a variety of cancers [1] . Tumor specific markers such as prostate-specific antigen [2], carcinoembryonic antigen [3], CA-15.3, and CA-27.29 have allowed for quick, non-invasive and inexpensive screening in large at risk populations in human medicine. Unfortunately, liquid biopsy techniques are rare in veterinary medicine.

Many platforms have been utilized to assess blood-based biomarkers for cancer. Traditionally, enzyme-linked immunosorbent assays (ELISAs) or polymerase chain reactions (PCR) have been utilized to detect specific biomarkers. However, cancers are multifactorial and very few cancers share biomarker expression between them or even within the same histology. Whole genome sequencing can cast a wider net to detect a variety of biomarkers in the blood, but this approach is costly and time consuming. ELISAs directed at a cancer surrogate, such as nucleosomes, can provide a rapid, cost effective and widely applicable approach for biomarker detection in the blood of both humans and veterinary patients.

Nucleosomes are small fragments of chromosomes released into the blood during cell death or white blood cell activation. These fragments consist of a histone octamer core with a short segment of DNA wrapped around it. Nucleosomes have demonstrated utility as epigenetic biomarkers for the detection and monitoring of a variety of human cancers including pancreatic, lung, and colorectal cancer [4–7]. Furthermore, plasma nucleosome concentrations have been shown to be predictive of outcome in patients with breast cancer. A multivariate analysis of 92 patients with breast cancer showed preoperative plasma nucleosome concentrations were as consistent as hormone receptor (HER2) status, lymph node status and tumor grade in predicting disease free survival [8]. Plasma nucleosome levels can also predict response to therapy in patients with non-small cell lung cancer (NSCLC). In a study of 134 patients with advanced NSCLC, treated with chemotherapy, those with significant decreases in nucleosome concentrations after their first cycle of chemotherapy had significant improvement on imaging after their 3^rd^ cycle. Those with insufficient decreases in plasma nucleosome concentrations were more likely to have stable or progressive disease and a significantly shorter progression free survival [9]. Circulating plasma nucleosomes have also been used to identify genome- and exome-wide cancer specific mutations as well as longitudinal changes throughout the course of treatment that can be used to capture clonal evolution and identify mechanisms of resistance [10, 11].

Plasma nucleosome concentrations are increased in a variety of diseases, including cancer. Letendre et al. published some of the earliest work describing the use of nucleosomes in dogs with trauma and sepsis in 2018 demonstrating that plasma nucleosome concentrations were positively correlated with a worse outcome in dogs with sepsis and trauma whereas cell free DNA did not demonstration a correlation [12, 13]. Additionally, similar to humans, increased plasma nucleosomes have been detected in cases of autoimmune disease and severe inflammation [12, 14–18]. Another study evaluating plasma nucleosome concentrations in dogs with acute and chronic gastrointestinal diseases found no statistical differences in nucleosome concentrations between the groups. However, dogs with acute and chronic GI disease tended to have slightly higher concentrations than the healthy control dogs. The one dog in this study with gastrointestinal lymphoma had a 10–20 fold increase in plasma nucleosome concentrations compared to the other groups [18].

Few studies exist defining the plasma nucleosome compartment in canine cancer. Recently, our group published data demonstrating increased plasma nucleosome concentrations in dogs with lymphoma and hemangiosarcoma [19–21]. In these studies, increased plasma nucleosome concentrations were found in the majority of cases with cancer, even in early stages of disease. The assay correctly identified 82% of hemangiosarcoma cases and 74% of lymphoma cases with 97% specificity. For dogs with hemangiosarcoma the assay was able to detect 67% of dogs with stage I, 76% of dogs with stage II and 90% of dogs with stage III disease. The assay was also able to distinguish LSA patients from healthy patients in 63% of stage I patients, 14.3% of stage II patients, 75.7% of stage III patients, 81.6% of stage IV patients, and 81.8% of stage V patients. Performance was also evaluated by immunophenotype and the threshold could distinguish LSA patients from healthy patients in 95.3% of B-cell LSA and 55.6% of T-cell LSA.

The goal of the current study was to expand upon that initial study to evaluate circulating nucleosome concentrations in dogs with a variety of common cancers. Understanding which cancer types are associated with increased nucleosome levels will not only expand the use of nucleosome screening but will also increase our understanding of nucleosomes and their role in cancer development.

## Results

**Fig. 1:**
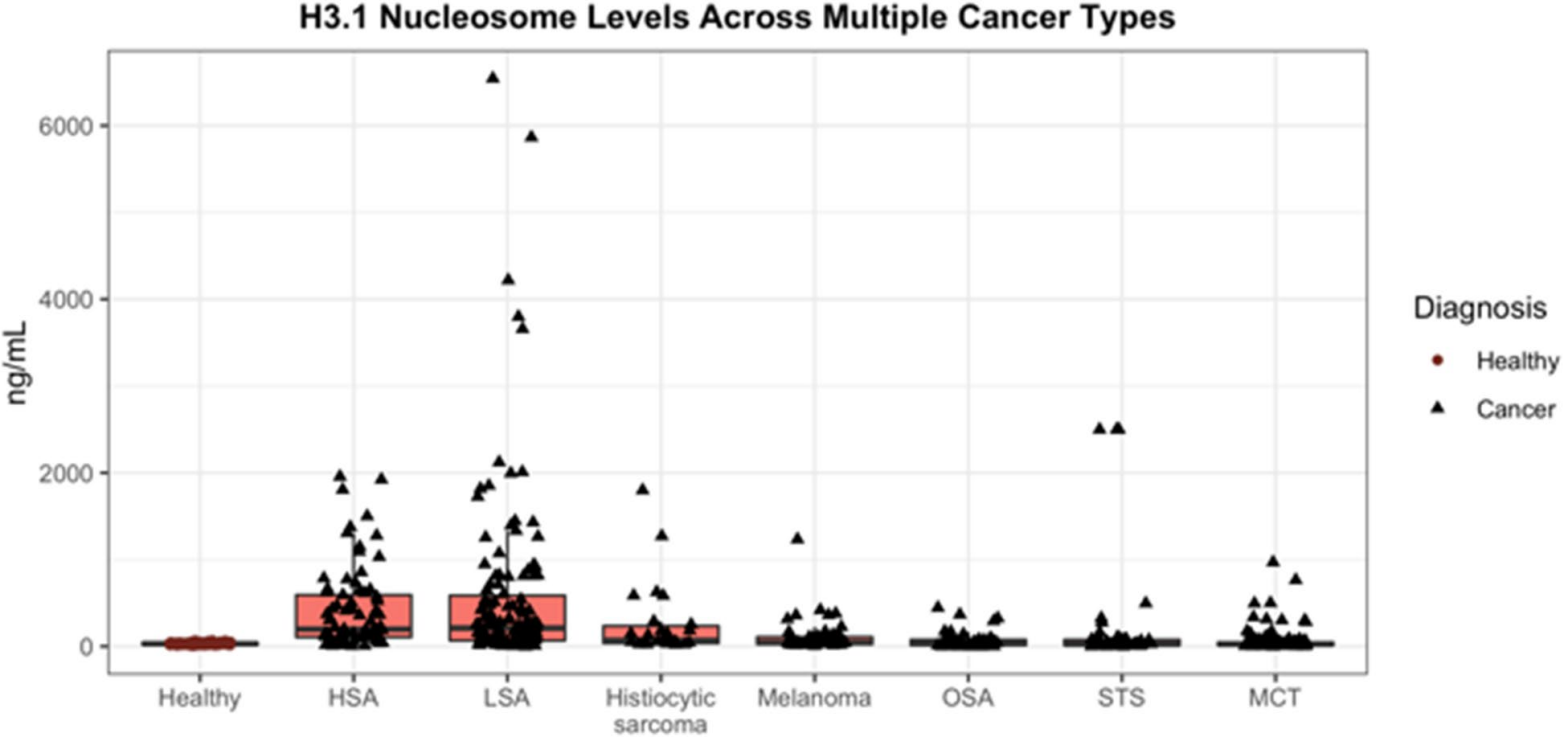
Box and whisker plot representing the various common cancers relative to the healthy cases. The y axis represents the plasma concentration of H3.1 nucleosomes in ng/mL and the x axis represents the different malignancies evaluated. (LSA- lymphoma, HSA- hemangiosarcoma, OSA- osteosarcoma, STS- soft tissue sarcoma, MCT- mast cell tumor, Hist Sarc- histiocytic sarcoma)

A total of 662 dogs were included in this study (528 dogs with cancer and 134 healthy dogs). The most common cancers evaluated included lymphoma (LSA; *n* = 126), Hemangiosarcoma (HSA; *n* = 77), osteosarcoma (OSA; *n* = 49), soft tissue sarcoma (STS; 51), malignant melanoma (*n* = 49), mast cell tumors (MCT; *n* = 126) and histiocytic sarcoma (*n* = 26) (Fig. 1). A variety of carcinomas (mammary (*n* = 1), pulmonary (*n* = 3), hepatocellular (*n* = 1), squamous cell (*n* = 3), urothelial cell (*n* = 3) and anal sac carcinomas (*n* = 3)) as well as a few miscellaneous tumors (multiple myeloma (*n* = 1), acute leukemias (*n* = 2), insulinoma (*n* = 1), nasal chondrosarcoma (*n* = 1), sertoli cell tumor (*n* = 1) and others) were also evaluated. These cases are described here however, due to the low case numbers of any specific histology, they are not included in the statistical analyses reported here.

Of the 528 dogs with cancer, 244 were spayed females, 15 were intact females, 237 were neutered males and 30 were intact males. Gender was unknown for 2 of the dogs. These dogs ranged in age from 1 – 19 years (median 9 years, mean 9.06 years) and ranged in weight from 5—74.5 kg (median 30.9 kg, mean 29.63 kg). The most commonly represented breeds included mixed breed dogs (*n* = 126), Labrador retriever (*n* = 58), golden retriever (*n* = 53) and boxers (*n* = 19).

A total of 134 healthy dogs were recruited for this study ranging in age from 10 months to 14 years (median 6 years). There were 61 spayed females, 4 intact females, 66 neutered males and 3 intact males. The most common breeds represented were mixed breed dogs (*n* = 28), Labrador retrievers (*n* = 15) and Australian cattle dogs (*n* = 10). Dogs were determined to be healthy based on results from a client questionnaire as well as a physical exam by the attending veterinarian. The median nucleosome concentration for all healthy dogs was 31.1 ng/mL (mean 32.07 ng/mL, SEM 1.118). For a specificity of 100% the cut off for the healthy range was set at 67.5 ng/mL (nucleosome range for all healthy dogs was 6.33—67.42 ng/mL). Neither age, neuter status, gender or size had an effect on nucleosome concentrations in either cohort of dogs [21] .

**Fig. 2:**
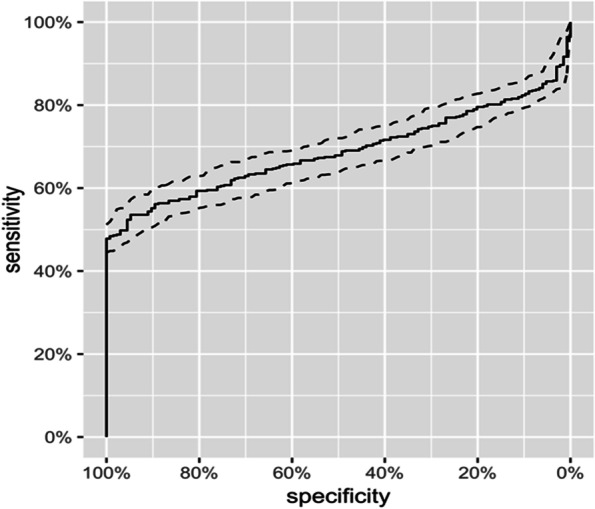
ROC curve demonstrating the AUC of 68.74% for all cancers when compared to healthy animals

When all cancer cases were considered together in comparison to healthy dogs the sensitivity of this assay was 49.8% with a specificity of 97% (Fig. 1). A receiver operator characteristic (ROC) curve was generated and the AUC was determined to be 68.74% (Fig. 2). The top 4 malignancies detected by the test included lymphoma, hemangiosarcoma, histiocytic sarcoma and malignant melanoma. The malignancies least likely to be detected using this assay were soft tissue sarcomas, osteosarcomas and mast cell tumors.

For the purposes of group comparisons, a single specificity of 97% was chosen and the sensitivity was then calculated using this parameter. The cancers with the highest sensitivities were lymphoma (76.98%), hemangiosarcoma (81.82%), visceral histiocytic sarcoma (61.9%) and malignant oral melanoma (50%). The cancers with the lowest sensitivity were cutaneous melanoma (0%), Mast cell tumors (19.05% all grades, grade 1: 33.33%, grade 2: 11.49% and grade 3: 34.62%) and primary bone histiocytic sarcomas (20%) (Table 1).

**Table 1:** Summary of the Sensitivity and specificity of plasma nucleosomes to detect a variety of canine cancers. (AUC- Area under the curve, Hist Sarc- Histiocytic Sarcoma, MCT- Mast cell tumor)

Cancer Histology	Number of cases	Increased Nu.Q® level	Sensitivity	Specificity	AUC
All Cancers	504	251/504	49.8%	97%	68.74%
Lymphoma	126	97/126	76.98%	97%	87.83%
Hemangiosarcoma	77	63/77	81.82%	97%	91.74%
Histiocytic Sarcoma	26	14/26	53.85%	97%	83.01%
Hist Sarc—bone	5	1/5	20%	97%	81.04%
Hist Sarc Visceral	21	13/21	61.9%	97%	83.48%
Melanoma (all)	49	21/49	42.86%	97%	70.36%
Melanoma [22]	42	21/42	50%	97%	75.05%
Melanoma (cutaneous)	7	0/7	0%	97%	42.22%
Mast Cell Tumor	126	24/126	19.05%	97%	43.68%
Grade 1 MCT	9	3/9	33.33%	97%	44.1%
Grade 2 MCT	87	10/87	11.49%	97%	11.49%
Grade 3 MCT	26	9/26	34.62%	97%	60.7%
Osteosarcoma	49	17/49	34.69%	97%	60.17%
Soft tissue sarcoma	51	15/51	29.41%	97%	48.19%

**Fig. 3:**
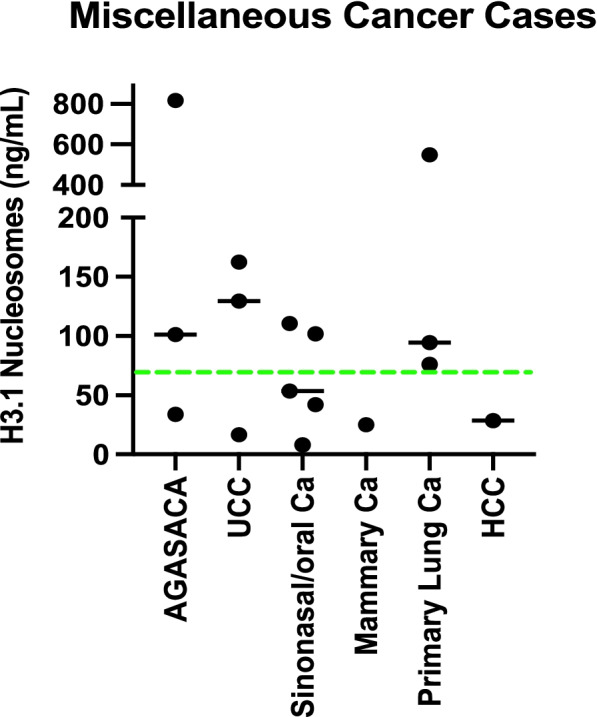
Graphic representation of plasma nucleosome concentrations in a variety of canine carcinoma cases. The green dotted line represents the normal cut off value of 67.4 ng/mL. Eight of the 16 carcinoma cases had increased plasma nucleosome concentrations. AGASACA- Apocrine gland anal sac adenocarcinoma, UCC- Urothelial Cell Carcinoma, Ca- Carcinoma, HCC- hepatocellular carcinoma

When considering the plasma nucleosome concentrations of dogs with a variety of carcinomas, eight of the sixteen cases had increased levels at the time of diagnosis. Two out of three of the anal sac apocrine gland adenocarcinomas, two of the three urothelial cell carcinoma cases and two of the 5 had sinonasal/oral carcinomas had increased plasma nucleosome concentrations at the time of diagnosis. The two dogs with increased levels were both diagnosed with oral squamous cell carcinomas. There were 3 dogs in this group with primary lung carcinomas, all of which had increased nucleosome concentrations and one each with mammary carcinoma and hepatocellular carcinoma that did not have increased nucleosome concentrations (Fig. 3).

Several other tumors were included in this study as well. Both dogs with acute leukemia had increased plasma nucleosome concentrations (lymphoblastic leukemia – 262.8 ng/mL, myeloblastic leukemia 423.5 ng/mL). We also saw increased plasma nucleosome concentrations in a dog with a sertoli cell tumor (118 ng/mL), a dog with a pharyngeal sarcoma (135.4 ng/mL), a dog with nasal chondrosarcoma (109.25 ng/mL) and a dog with multiple myeloma (71.7 ng/mL). There were three cases that did not have increased plasma nucleosome concentrations including a dog with an undifferentiated splenic sarcoma (16.4 ng/mL), a dog with insulinoma (8.5 ng/mL) and a dog with multilobular osteochondrosarcoma (20.3 ng/mL).

Detailed evaluations of plasma nucleosome concentrations in dogs with lymphoma and hemangiosarcoma by stage, location or phenotype have been described elsewhere [20, 21]. For the cases with melanoma there were two main groups. The first represents cutaneous melanoma of haired skin which are often considered benign (*n* = 7) and those in the oral cavity (*n* = 42) which are often malignant. The median plasma nucleosome concentration for the dogs with cutaneous melanoma was 24.8 ng/mL (mean 27.6 ng/mL, range 7.3–43.9) and the median size of these tumors was 3 cm (mean 4.7 cm, range 1.8–12 cm) (Fig. 3). The median plasma nucleosome concentration for dogs with oral melanoma was 60.0 ng/mL (mean 130.422, range 14.0–1234.5 ng/mL) and the median size of these tumors was 4 cm (mean 4.5 ng/mL, range 1.5–15 cm) (Fig. 3). While the highest nucleosome concentration was seen in the largest tumor (1234.5 ng/mL, 15 cm), size of the lesion was not associated with an increased nucleosome concentration for those cases for which size was available. Mitotic index was not available for evaluation.

**Fig. 4:**
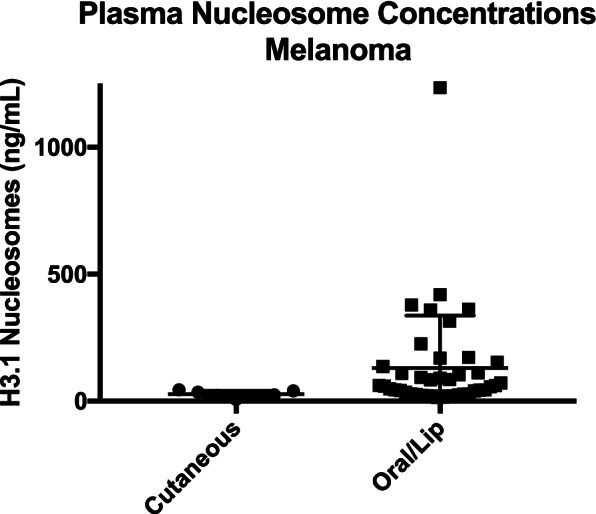
Dot plot expressing mean with SEM of plasma nucleosome concentrations (ng/mL) for melanoma cases by location

**Fig. 5:**
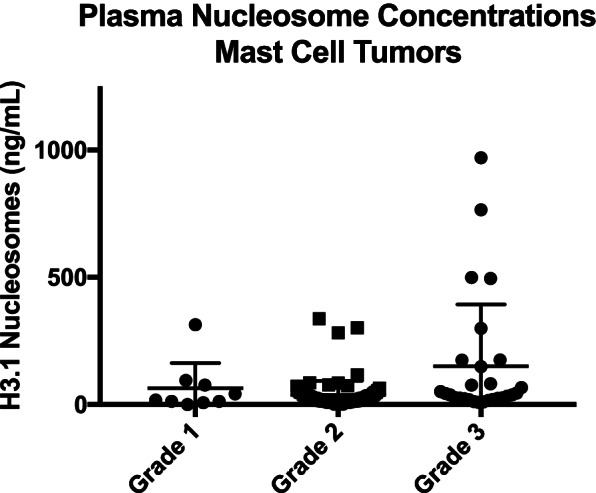
Dot plot expressing mean with SEM of plasma nucleosome concentrations from dogs with mast cell tumors according to grade

Mast cell tumors were divided by recorded grade. All tumors in the DCTD biobank were categorized according to the Patnaik 3 tier grading scheme. Grade 3 tumors had the highest median and mean plasma nucleosome concentrations (38.2 and 149.7 ng/mL respectively) followed by grade 1 tumors. Grade 2 tumors had the lowest nucleosome concentrations of the group (Fig. 4, Table 2). When considering low grade mast cell tumors along with those of unknown grade (*n* = 4), the grade 3 mast cell tumors had a significantly higher nucleosome concentration than both the low grade tumor cases and the healthy dogs (*p* = 0.005) (Table 3, Fig. 5). Tumor size and stage of disease was not available for comparison. Follow up information regarding overall survival was not available for the dogs with increased plasma nucleosome concentrations.

**Table 2:** Nucleosome concentrations by histology. The *p*-values represent the Wilcoxon Rank Sum (Mann Whitney U) test for each group against the control group, which in this case is Healthy. (HSA- hemangiosarcoma, LSA- Lymphoma, OSA- Osteosarcoma, STS- Soft Tissue Sarcoma, MCT- Mast cell tumor)

H3.1 (ng/mL)​	n​	Minimum​	Maximum​	Median​	Mean​	p​
HSA​	77​	6.54​	1956.90​	198.30​	414.62​	**6.33E-24​**
LSA​	126​	0.10​	6544.00​	211.05​	570.87​	**5.62E-26​**
OSA​	49​	0.10​	446.00​	43.20​	72.70​	**0.0354​**
STS​	51​	0.10​	2500.00​	25.09​	200.07​	0.7043​
MCT​	126	0.10​	969.45​	24.65​	64.21​	0.0784​
MCT Grade unk	4	11.02	174.58	83.77	88.29	0.8341
MCT Grade 3	26	8.39	969.45	38.21	149.74	0.0849
MCT Grade 2	87	3.10	337.23	22.67	37.52	**0.0029**
MCT Grade 1	9	0.10	313.81	18.26	64.41	0.5579
Melanoma​	49​	7.28​	1234.50​	44.59​	115.73​	**2.53E-05​**
Histiocytic sarcoma​	26	21.81​	1800.00​	69.59​	261.22​	**1.06E-07​**
Healthy​	134​	6.33​	67.42​	31.10​	32.07​	1​

**Table 3:** Comparing MCT Grade 3 to MCT Grades 1, 2 and unknown gives a *p*-value of 0.0050, which is significant

H3.1 (ng/mL)​	n	Minimum	Maximum	Median	Mean	p
MCT Grades 1 2 X	100	0.1	337.23	21.688	41.96727	1
MCT Grade 3	26	8.3887	969.45	38.2075	149.73514	**0.0050**
Healthy	134	6.332834	67.41702	31.09814	32.07375	**0.0050**

**Fig. 6:**
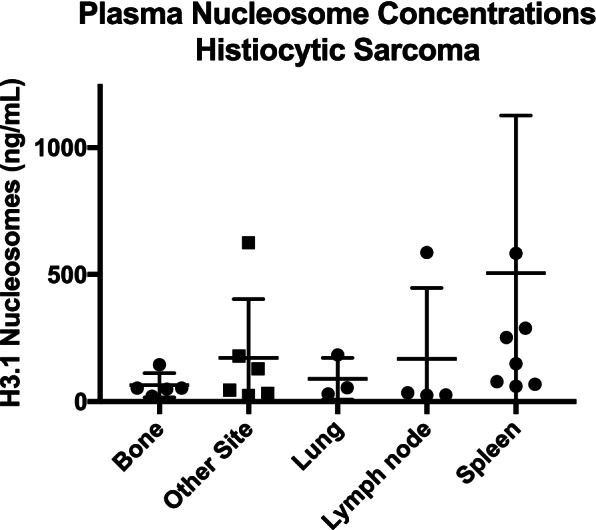
Dot plot of mean with SEM for plasma nucleosome concentrations of histiocytic sarcoma based on location

Overall, visceral histiocytic sarcomas as a group had increased plasma nucleosome concentrations similar to lymphoma and hemangiosarcoma. Those cases involving the spleen had the highest median plasma nucleosome concentrations. Histiocytic sarcomas involving the bone had the lowest median plasma nucleosome concentrations (Fig. 6) [21]. Stage and size of the tumor was not available for comparison.

Of the 50 soft tissue sarcomas evaluated in the study, 15 had increased plasma nucleosome concentrations. Six of these 15 cases were located in the skin/subcutis. Five were primary splenic sarcomas (non-hemangiosarcoma). Two were malignant peripheral nerve sheath tumors. One was ocular, one was primary renal and two had no primary site listed. Tumor size, grade and stage were not available for comparison. Of the 50 STS cases, 18 were primary skin/subcutis tumors with 33% (6/18) having increased plasma nucleosome concentrations. Ten of the cases were primary muscle STS and none of them had increased plasma nucleosome concentrations. Seven of the cases were primary splenic non-hemangiosarcoma sarcomas with 71.4% (5/7) having increased plasma nucleosome concentrations. Five were primary bone non-osteosarcoma sarcomas and none of these cases had increased plasma nucleosome concentrations. There was one case each of primary renal, auricular and ocular sarcomas. Seven of the STS cases had no reported location.

Just under 35% (17/49) of the osteosarcoma cases demonstrated increased plasma nucleosome concentrations at diagnosis. Interestingly, the dog with the highest plasma nucleosome concentration in the osteosarcoma group (446 ng/mL) was the one dog that presented with metastatic disease at diagnosis (Tables 1 and 2). When evaluated based on location, there were 9 cases with primary femur OS, 33% (3/9) had increased plasma nucleosome concentrations. One of these dogs was the one with metastatic disease. Sixteen were primary humeral OS cases and 37.5% (6/16) had increased plasma nucleosome concentrations. Eleven cases had primary radius OS and only one (9%) had increased plasma nucleosome concentrations. Eleven cases had primary tibial OS and 4 (36%) had increased plasma nucleosome concentrations.

## Discussion

Nucleosome levels were evaluated in plasma samples from patients with seven of the most common canine cancers. The dogs with the most frequent elevations in plasma nucleosome concentrations were dogs with hemangiosarcoma and lymphoma, consistent with previous publications [20, 21]. Other cases with frequent elevations in plasma nucleosome concentrations include those with histiocytic sarcoma and oral malignant melanoma.

In this cohort, the H3.1 ELISA was able to correctly identify 174 or 229 cases of systemic cancer (76%) including lymphoma, hemangiosarcoma and histiocytic sarcoma. Overall, the test was able to correctly identify 49.8% of all of the cancers evaluated in this study. This compares favorably with other similar tests in the veterinary and human space. In the PATHFINDER study published by GRAIL, the Galleri test had a positive predictive value of 49% of the 50 cancers studied in humans [23] . In the veterinary space, the CANDID study was able to detect 54.7% of all the cancers they studied in that cohort and 85.4% of lymphoma, hemanogiosarcoma and osteosarcoma cases [24] .

Plasma nucleosome concentrations are not specific for cancer. In humans, a variety of other diseases have been shown to cause increases in plasma nucleosome concentrations such as diabetes mellitus type 2, pediatric acute respiratory distress syndrome, chronic kidney disease, pancreatitis, trauma, and COVID19 infections among other diseases [25–27] [26, 28–30]. In dogs, increased plasma nucleosome concentrations have been noted in dogs with sepsis, after trauma and in cases of hemolytic anemia [12–15, 31] . Because these diseases are often associated with inflammation and frequent cell death, it is not surprising that plasma nucleosome concentrations are increased in these cases. Very little has been published in the literature regarding elevated plasma levels of nucleosomes in diseases other than trauma or inflammation in canines. Our group is currently working to evaluate many other common chronic and acute diseases in canines.

Not surprisingly, the majority of dogs with visceral disease had increased plasma nucleosome concentrations. However, only one of the cases with primary osseous histiocytic sarcoma had increased plasma nucleosome concentrations. This is in contrast to the fact that 100% of primary osseous hemangiosarcomas had increased plasma nucleosome concentrations in one study [21]. In this study, only 34.7% of dogs with osteosarcoma had increased plasma nucleosome concentrations at diagnosis as well. Though osteosarcoma in dogs is often an aggressive tumor with a rapid growth rate, the local nature of this disease at the time of diagnosis is most likely part of the reason why so few of these cases have increased nucleosome concentrations. Interestingly the dog diagnosed with osteosarcoma that had the highest elevations in nucleosome concentrations (446 ng/mL) was the only dog in the osteosarcoma cohort that had evidence of metastatic disease, suggesting that as this disease becomes more systemic it may be more likely correlated with elevations in plasma nucleosome concentrations. Additional cases of metastatic disease are needed to determine the value of plasma nucleosome concentrations as a predictor of metastatic disease in dogs with osteosarcoma as well as other cancers.

Overall, the assay was only able to correctly predict 19% of the cases with mast cell tumors. Dogs with grade 1 tumors were detected 33% of the time and dogs with high grade tumors were detected 34% of the time, whereas grade 2 tumors were only detected 11.5% of the time. It is unclear from the patient information available why there is a difference between grade 2 tumors and the other grades. Interestingly, the high grade tumors had a significantly higher mean nucleosome concentration compared to low grade tumor cases and healthy dogs. The samples used for this particular study were graded using the older Patnaik grading scheme. Additional work is needed to see if this same difference holds true when the newer two-tier grading system is applied. Unfortunately, we do not have the outcome data associated with this group and cannot determine whether those cases with increased plasma nucleosome concentrations have a better or worse prognosis.

Interestingly, in this cohort of samples none of the benign cutaneous melanomas had increased plasma nucleosome concentrations, while just over half of the malignant oral melanomas demonstrated increased plasma nucleosome concentrations. This finding highlights the possibility of using nucleosome levels to discriminate between benign and malignant processes in dogs with melanoma. All of the malignant melanomas in this study were oral in origin. Oral tumors, in particular melanomas and squamous cell carcinomas, often have significant inflammation associated with them. This may be adding to the plasma nucleosome concentrations in the 21/42 cases of oral melanoma and 2 oral squamous cell carcinomas described in this study. Additional cases from other commonly malignant locations such as the ungual process or the perianal region need to be evaluated to determine if this trend holds true for all cases of malignant melanoma.

Similarly to what has been noted in humans, the three cases with primary lung carcinomas had elevations in plasma nucleosome concentrations [32–35]. Two of the three dogs with anal sac tumors also had significant elevations in plasma nucleosome concentrations. The one dog with a plasma nucleosome concentration over 800 ng/mL had stage I disease that was treated with surgery demonstrating that this may also be a useful tool for dogs with early stage anal sac neoplasia. Two of the 5 cases of oral/sinonasal carcinomas had increased nucleosome concentrations. Given the local nature of these diseases, we did not expect to see elevations in any of these cases. However, both of these cases were squamous cell carcinomas. These tumors tend to have a large inflammatory component that may be responsible for the elevations of plasma nucleosome concentrations seen here. It was also surprising to see increased nucleosome concentrations in 2/3 of the cases with urothelial carcinoma. Again, these cases had local disease only. It was not surprising to see increased nucleosome concentrations in the one case of multiple myeloma given that it is a systemic tumor and a tumor of plasma cells. However, the fact that the stage III nasal chondrosarcoma, which tends to be slow growing, displayed elevations in plasma nucleosomes was unexpected. Local inflammation at the site of the tumor may play a role here and additional cases of sinonasal tumors as well as non-melanoma oral malignancies are being enrolled. Several cases did not have increased plasma nucleosome concentrations. These include the cases of insulinoma, mammary carcinoma, MLO, undifferentiated splenic sarcoma and hepatocellular carcinoma did not demonstrate elevations in plasma nucleosome concentrations. This may be due to the low disease burden or the slow cellular turnover rate associated with most of these tumors. Additional cases of non-hemangiosarcoma splenic masses are being actively recruited to better understand these diseases.

## Conclusions and future directions

In summary, although the majority of information published regarding plasma nucleosome concentrations in dogs with cancer have been centered around lymphoma and hemangiosarcoma, this test is likely useful for detecting other forms of cancer as well. The test performs best for tumors that are systemic (higher metastatic rate) and for those that have a high cellular turnover rate. This is consistent with the observation that nucleosomes are released into the plasma at a higher rate when there is a rapid cellular turn over and high cellular death rates. This is also true for non-cancerous diseases including severe infections or inflammation. Additional studies are ongoing to determine how noncancerous concomitant diseases affect the plasma nucleosome compartment in dogs.

Evaluation of H3.1 plasma nucleosome concentrations using a low-cost simple ELISA test requiring low blood sample volume and may also provide additional information regarding the overall health and well-being of companion dogs. While this test was only able to detect approximately half of the cancers it tested for, it is well positioned as a companion test to other wellness tests and has the potential to provide valuable additional information that can inform the clinical decision-making process. Additional assays are needed to improve the sensitivity of all liquid biopsy techniques in both the human and veterinary spaces.

## Methods

### Sample collection and processing

All animal studies were approved by the Texas A&M University Institutional Animal Care and Use Committee (36 2019–0211 and AUP# 2017–0350). A detailed characterization of the participants has been included in the results section. The lymphoma and hemangiosarcoma cases were previously described in two publications from 2021 [20, 21]. These cases were included here in order to generate a broad AUC and sensitivity and specificity of this assay for the most common canine cancers. Canine plasma samples from the National Cancer Institute Division of Cancer Treatment and Diagnosis (NCI-DCTD) biorepository and from active patients or healthy volunteers at the Texas A&M University Small Animal Teaching Hospital were purchased or collected with owner consent, respectively, for this study. A minimum of 0.5 mL of plasma was collected from each patient. Dogs were fasted for a minimum of 4 h before blood collection. Samples were drawn from a peripheral or jugular vein into a K2 EDTA tube (BD Vacutainer, Franklin Lakes, NJ) and centrifuged at 3000 g for 10 min within 1 h of collection (Clinical 100 centrifuge, VWR, Radnor, PA). The plasma samples were labeled and frozen at -80 °C until samples were run in batches.

All samples were tested using the Nu.Q™ H3.1 assay (Belgian Volition, SRL, Isnes, Belgium). This enzyme-linked immunosorbent assay (ELISA) contains a capture antibody directed at histone 3.1 and a nucleosome specific detection antibody [36]. Frozen samples were thawed and allowed to come to room temperature for at least 30 min prior to analysis. Assays were performed according to the manufacturer’s protocol. Briefly, a standard curve was generated using the positive control stock (recombinant H3.1 nucleosomes) provided. Samples were vortexed and centrifuged at 11,000 × g for 3 min at 4 °C. HSA samples were diluted threefold to ensure that they could be measured within the detection limits of the assay. The nucleosomes were bound to the capture antibody and the plates were washed 3 times using 1 × wash buffer. Twenty microliters of each diluted sample were pipetted in duplicate into wells on the 96 well plates. Next, 90μL of the assay buffer was added to each well. The plate was covered with sealing film and incubated on an orbital shaker for 2.5 h at 700 rpm. Plates were then emptied and washed 3 times using 1 × washing buffer. Next, 100 uL of the detection antibody was added to each well, the plate was resealed and incubated for 1.5 h on the orbital shaker. The plates were then washed as described above. Streptavidin HRP conjugate was incubated for 30 min in each well and washed before applying the colorimetric substrate solution and incubating the plates in the dark for 20 min. A stop solution was added to the wells and the plates were read on a plate reader at 450 nm (BioTek Synergy H1 plate reader, BioTek Instruments, Winooski, VT). The standard curve was linearized and fitted to a 4-parameter logistic curve using statistical software (Graphpad Software, version 8, San Diego, CA). Two kit controls (KC) are included for each plate. KC1 has a value near the lower level of detection and KC2 has a value near 200 ng/mL. The kit control values must fall within a predetermined range for the plate to be used to limit interplate variability.

### Data processing of H3.1 plasma measurements

Duplicate raw values recorded by the lab were expressed in optical density (OD) values, that is the color intensity related to the quantity of the biomarker present in the sample. The transformation of OD values was done using the standards with assigned concentrations, allowing the preparation of quantification curves for a calculation of relative concentration. The final record for each subject is a mean of duplicate concentration measurements for each sample tested on each biomarker. Any concentration values recorded with a co-efficient of variation [37] percentage above [20%] were rejected and the analysis repeated. The standard curve was linearized and fitted to a 4-parameter logistic curve using statistical software (Graphpad Prism Software, version 9, San Diego, CA, USA, www.graphpad.com). Concentration values calculated in this way are expressed in ng/ml.

The dynamic range is defined as the usable range of an assay between the upper and lower limits of quantification (LLOQ = lower, ULOQ = upper). Where a reading is above the ULOQ, there is a procedure for the serum sample to be diluted and measured again in relation to the standard curve to provide a good quantity of biomarker in the tested serum. Where a reading is below the LLOQ, the value for that sample is set to a concentration of zero.

### Outliers and missing values

No samples were removed from the analysis if they were calculated to be outliers for any variable. Subjects with missing values for a variable used for a calculation were removed from that particular calculation only, where the result is undefined for missing values. Subjects with missing values for variables other than those used in the calculation being performed were not removed.

### Data analysis

Descriptive statistics for the patient populations were performed using Microsoft Excel for Mac (v. 16.16.27, 2016). A Shapiro–Wilk test was performed, and the data was determined to not be normally distributed (no group had a test value above 0.1) For this reason, nonparametric tests were utilized for data comparisons. For data sets containing only two cohorts, such as the healthy controls versus all mast cell tumor cases, a Wilcoxon rank sum test was used to determine whether there is a statistical difference between the orderings of the two sets (with a null hypothesis that the probability that a randomly drawn observation from one group is larger than a randomly drawn observation from the other group is equal to 0.5 against an alternative that this probability is not 0.5). For data sets where multiple conditions were compared, such as when all cancers were considered, a Kruskal–Wallis test for repeat measures with a Dunn’s multiple comparisons test was performed using GraphPad Prism Software version 9, San Diego, California USA, www.graphpad.com.

To assess the diagnostic performance of the Nu.Q™ H3.1 assay, Receiver Operator Charatceristic (ROC) curves were calculated, along with the Area Under the ROC Curve (AUC). The sensitivity was calculated for each level of specificity. To make it easy to compare different types of cancer, a standard sensitivity of 97% was used, and it was noted that this was not far from the cut-off for whcih Youden's Index (J = sensitivity + sepcificity -1) was maximized (53.57% sensitivity at 94.78% specificity).

Wilcoxon rank sum tests, ROC curves and specificity/sensitivity calculations were only performed on cancer groups for which there were at least 20 cases. These calculations were performed using R version 3.4.3 and the pROC package [38, 39].

## Data Availability

All relevant data are within the paper.

## Abbreviations

DNA: Deoxyribose Nucleic Acid
PSA: Prostate Specific Antigen
CEA: Carcinoembryonic Antigen
ELISA: Enzyme Linked Immunosorbent Assay
PCR: Polymerase Chain Reaction
NSCLC: Non Small Cell Lung Cancer
LSA: Lymphoma/Lymphosarcoma
OSA: Osteosarcoma
STS: Soft tissue Sarcoma
MCT: Mast Cell Tumor
SEM: Standard Error of the Mean
ROC: Receiver Operator Characteristic Curve
NCI-DCTD: National Cancer Institute Division of Cancer Treatment and Diagnosis biorepository
EDTA: Ethylenediaminetetraacetic acid
g: Relative Centrifugal Force
C: Celsius
ANOVA: Analysis of Variance
